# Utilizing bioprinting to engineer spatially organized tissues from the bottom-up

**DOI:** 10.1186/s13287-024-03712-5

**Published:** 2024-04-08

**Authors:** Yichen Zhan, Wenbin Jiang, Zhirong Liu, Zhenxing Wang, Ke Guo, Jiaming Sun

**Affiliations:** 1 Department of Plastic Surgery, Union Hospital, Tongji Medical College, Huazhong University of Science and Technology, Wuhan, 430022 China; 2 Wuhan Clinical Research Center for Superficial Organ Reconstruction, Wuhan, 430022 China

**Keywords:** Bioprinting, Bottom-up, Tissue engineering, Assembly, Building blocks

## Abstract

In response to the growing demand for organ substitutes, tissue engineering has evolved significantly. However, it is still challenging to create functional tissues and organs. Tissue engineering from the ‘bottom-up’ is promising on solving this problem due to its ability to construct tissues with physiological complexity. The workflow of this strategy involves two key steps: the creation of building blocks, and the subsequent assembly. There are many techniques developed for the two pivotal steps. Notably, bioprinting is versatile among these techniques and has been widely used in research. With its high level of automation, bioprinting has great capacity in engineering tissues with precision and holds promise to construct multi-material tissues. In this review, we summarize the techniques applied in fabrication and assembly of building blocks. We elaborate mechanisms and applications of bioprinting, particularly in the 'bottom-up' strategy. We state our perspectives on future trends of bottom-up tissue engineering, hoping to provide useful reference for researchers in this field.

## Introduction

Increasing prevalence of organ failure is a huge burden for public health nowadays, while shortage of available grafts intensifies this problem. Tissue engineering, an important discipline targeting at using biomaterials and cells to restore, maintain and enhance function of tissues and organs, has drawn great interest because of its potential in creating artificial organs. Since the emerging of the tissue engineering concept in the 1980s [1], efforts have been made in this field for decades and artificial tissues such as cartilage, bone, skin, vessel, adipose tissue, muscle and tendon have been created in laboratory [2–4], proving the feasibility of engineering tissues.

**Fig. 1:**
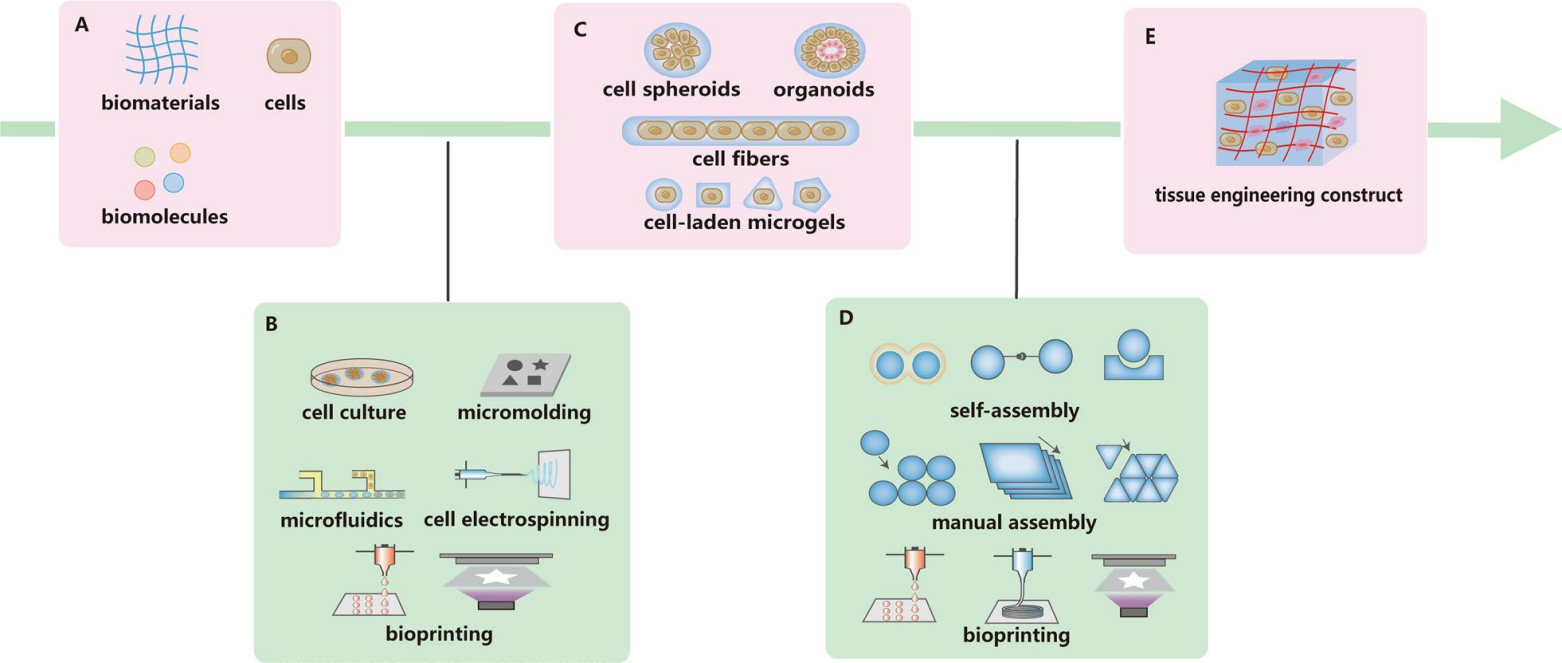
Workflow of bottom-up tissue engineering. In bottom-up tissue engineering, biomaterials, cells and biomolecules (to support the survival of cells, direct the differentiation or other functions) (**A**) are the basic components to fabricate building blocks. The structure and size of building blocks depends on its application and fabrication method (**B**), while cell spheroids, organoids, cell fibers or microgels of specific shapes (**C**) are commonly used. Then the building blocks are assembled through self-assembly, manual assembly or bioprinting (**D**) to an organized artificial tissue (**E**)

Regarding the roadmap of tissue engineering, a fundamental dichotomy exists in the strategic approaches known as 'top-down' and 'bottom-up' [5]. The top-down strategy involves the seeding of cells onto biomaterial scaffolds, typically supplemented with growth factors to regulate cell behavior and stimulate extracellular matrix production. In top-down strategy, cells are supposed to migrate and proliferate to fill the scaffolds. However, low cell density and uneven cell distribution resulting from the direct seeding of cells make the expansion of cells difficult. And this strategy can’t recreate the microstructure of tissues and organs. In contrast, the 'bottom-up' strategy centers on the systematic fabrication and assembly of basic building blocks constituting tissues and organs (shown in Fig. 1). Cell-laden building blocks ensure the even distribution of cells, thus increase the cellular viability and promote the formation of artificial tissues [6, 7]. The building blocks are also the basic units of microstructure of tissues. Moreover, the microvascular network can be designed in the bottom-up strategy without adding obstacle for cell growing, while the microvascular design in top-down strategy increases the intricacy of the scaffold and hinders the cell expansion [8]. Therefore, the bottom-up strategy is advantageous because of even cell distribution, microstructure replication, and microvascular network design.

A variety of techniques have been utilized to construct tissues and organs from the ‘bottom-up’, including microfluidics, micromolding, self-assembly, 3D bioprinting, electrospinning and so on. Among these techniques, bioprinting is free-form and flexible in the aspect of producing 3D structures. Besides, this automated technique can be precisely controlled to create spatially organized tissues with complex architecture. And assorted printing systems and bioinks designed for bioprinting have made it a versatile approach in biofabrication.

Several reviews have already summarized fabrication and assembly techniques of modular tissue engineering [5, 8–11]. In this review, we focus on applications of bioprinting in bottom-up tissue engineering, especially its capability of mimicking physiological complexity to form spatially organized tissues. First, we make a brief description of techniques applied in manufacturing building blocks. Then we provide a thorough review of bioprinting as a useful tool for the assembly of building blocks, including inkjet bioprinting, extrusion-based bioprinting and light projection bioprinting. Finally, we depict possible trends of future research and yet unsolved challenges of bioprinting functional tissues.

## Mimicking the physiological complexity of living tissues

Facilitated by the intricately organized complexity inherent in physiological systems, organs and tissues function effectively [12]. Morphologically, the physiological complexity of living tissues and organs is organized in two aspects, namely basic units and various cell types in an organ [13]. Firstly, many organs are made up of repetitive units [14]: for example, livers have lobules, bones contain osteons, muscles consist of fibers, and the lungs feature alveoli (see Table 1). An adequate amount of repeating functional units ensures the durability and stability of organ function, while also prompting the uniform distribution of cells and extracellular matrices in living tissues. Secondly, living tissues exhibit a specific spatial architecture, with various cells integrated into a cohesive whole [15]. These two aspects of physiological complexity are crucial for the optimal organ function, while its disruption can compromise organ function. In tissue engineering from the bottom up, spatially arrangement of building blocks aims to mimic this physiological complexity.

**Table 1:** Repetitive units in living tissues or organs

Tissues/organs	Repetitive units	Structure	Function	References
Liver	Hepatic lobule	Hexagon or triangle island of hepatocytes interspaced by sinusoids	Metabolism	[16]
Kidney	Nephron	Composed of renal tubule and renal corpuscle, a tuft of capillary surrounded by bowman’s capsule	Filtering blood and regulate body fluid	[17, 18]
Lung	Pulmonary alveolus	Cavities of alveolar membrane surrounded by capillaries	Pulmonary gas exchange	[4]
Bone	Osteon	Cylindrical structures of concentric layers of bone tissues	Stability of bone and facilitating nutrient exchange	[19]
Skeletal muscle	Myofibril	Tubular fiber of long elastic proteins wrapped by sarcoplasmic reticulum	Muscle contraction	[20, 21]
Myocardium	Sarcomere	Aligned fibers between intercalated discs	Muscle contraction	[22]

**Fig. 2:**
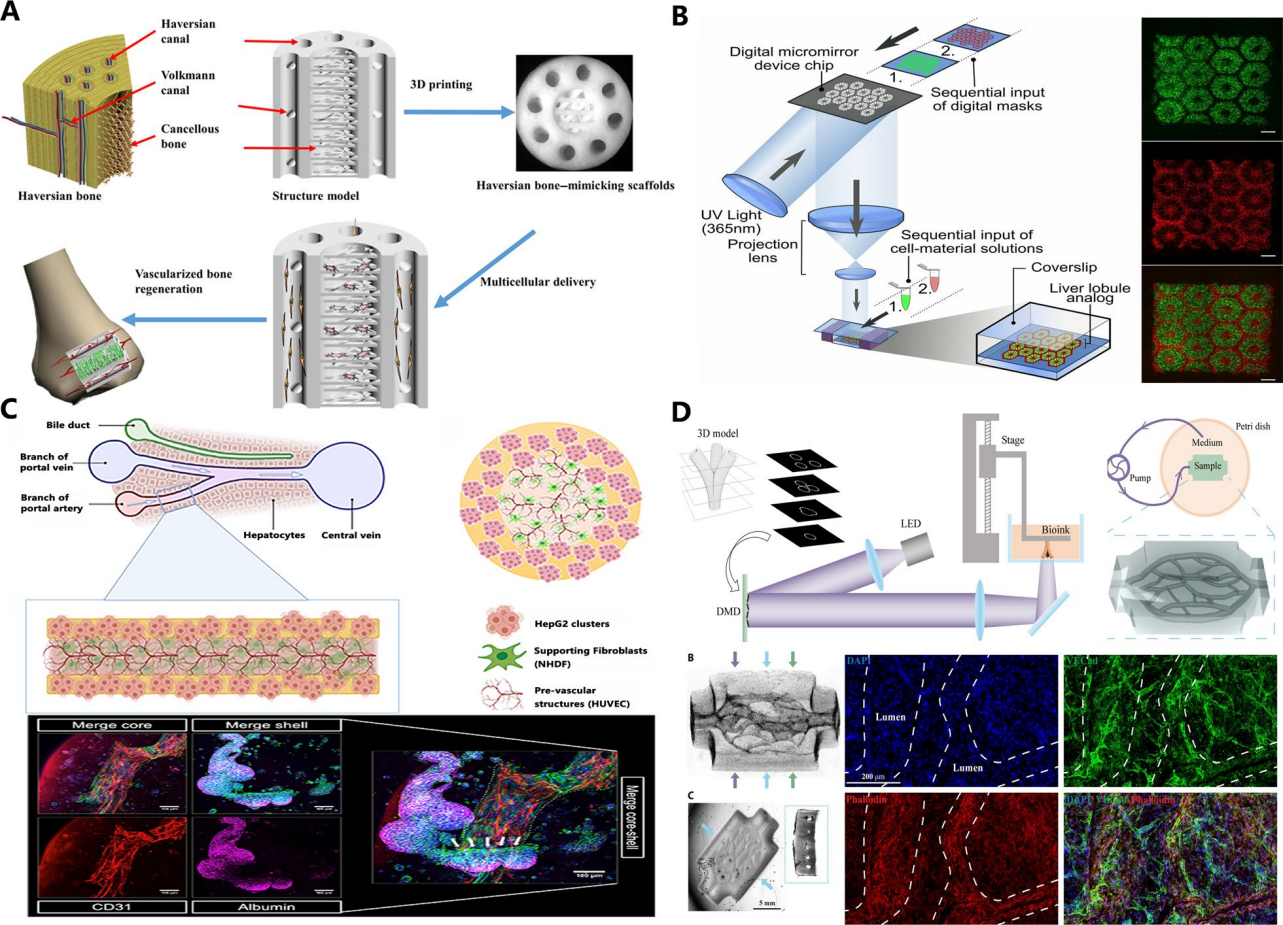
Mimic fundamental units of living tissues. **A** Haversian bone–mimicking bioceramic scaffolds (from Zhang M, Lin R, Wang X, et al. Sci Adv. 2020;6(12), reproduced under the terms of Creative Commons Attribution NonCommercial License 4.0). **B** Microscale hexagonal model mimicking liver lobules (Reproduced with permission, Ma X, Qu X, Zhu W, et al. Proc Natl Acad Sci. 2016;113(8):2206–2211). **C** Core–shell bioprinted liver sinusoid-like model (from Taymour R, Chicaiza-Cabezas NA, Gelinsky M, Lode A. Biofabrication. 2022;14(4): 045019, reproduced under the terms of Creative Commons Attribution 4.0 license). **D** High cell density tissue with vascular network (from You S, Xiang Y, Hwang HH, et al. Sci Adv. 2023;9(8):eade7923, reproduced under the terms of Creative Commons Attribution license)

Several studies have reported the production of building blocks that resemble the repetitive units of living tissues. Zhang et al. constructed Haversian bone–mimicking bioceramic scaffolds by 3D printing (Fig. 2A). The scaffolds contain repeating canals, mimicking the Haversian canals and Volkmann canals in osteon. In addition, bone marrow derived mesenchymal cells (BMSCs) and human umbilical vein endothelial cells (HUVECs) can be delivered through the canals of scaffold, promoting osteogenesis and vascularization [19]. Ma and his colleagues used digital light projection (DLP) bioprinting to fabricate a microscale hexagonal model to mimic liver lobules (Fig. 2B). After co-culturing hepatic cells and epithelial cells, the model could recapitulate structural feature and metabolic function of liver [16]. Taymour et al. built a liver sinusoid-like model through core–shell bioprinting (Fig. 2C). The model consisted of a core compartment containing pre-vascular bioinks and a shell compartment containing hepatocytes, resembling hepatic sinusoids, a fundamental structure of liver [23]. Fabricating small vascularized units is a strategy to construct a well-vascularized large tissue. It requires high resolution processing technique. You et al. reported that they used refined light projection bioprinting technique to fabricate tissue with vascular network with diameter of 250–600 μm (Fig. 2D), which supported living cells at a high density of 4*10^7^/ml[24].

Besides mimicking repetitive units of organs, it is also significant to reconstruct the spatial organization of cells, extracellular matrices and biomolecules in living tissues. Cardiac systolic and diastolic function rely on the hierarchical organization of myocardium, fibers in extracellular matrices and the synchronization of muscle and valves. Therefore, replicating the spatial arrangement of heart is critical for the construction of functional artificial heart. Lee et al. reconstructed hierarchical collagen fibers, ventricle consisting of collagen wall and cardiomyocytes and tri-leaflet heart valve through bioprinting. The 3D printed heart in their study demonstrated robust blood ejection capacity similar to human heart [25]. Another example of the significance of spatial organization to function is the cartilage. Extracellular matrices of cartilage contain bush-like lubrication complex which is assembled from glycosaminoglycans, hyaluronic acids, phospholipid and lubricin. These bush-like lubrication complexes are anchored to collagen fiber through fibronectin [26]. Xie et al. proposed that mimicking the structure of the brush-like complex can confer lubrication property and they designed the backbone and brunch of the polymer in hyaluronic acid hydrogel to restore the function of cartilage in a rat osteoarthritis model [27].

## Manufacturing building blocks for modular tissue engineering

The first step of constructing artificial tissues from the bottom-up is the fabrication of building blocks. In this section, we review several representative manufacturing methods. Based on their ability to govern the configuration of the fabricated constructs, these methods are described in the order of 3D culture, emulsification, microfluidics, cell electrospinning, micromolding and bioprinting [7]. 3D culture and emulsification are not able to produce constructs of specific shape and size. Microfluidics and cell electrospinning can produce uniformly sized sphere particles or fibers. Micromolding and bioprinting afford precise control over both the shape and size of the fabricated construct. We compare those fabrication methods of building blocks (see Table 2) and we highlight the application of bioprinting.

**Table 2:** Representative fabrication methods for building blocks

Fabrication method	Building blocks	References	Advantages	Disadvantages
3D cell culture	Organ buds from endothelial cells and MSCs cultured with tissue-specific progenitors or relevant tissue samples	[28]	Generative potential Promote vascularization	Limited productivity Strict culture condition
	Mouse or human ISC-derived organoids in PEG hydrogels	[29]		
	Printed hMSCs cultured in alginate microgel	[30]		
	HiPSCs aggregates suspended in ECM solution to form embryoid bodies	[31]		
Emulsification	Hollow spherical cell aggregates in gelatin microbeads generated by emulsification in oil bath	[32]	Easy to operate Moderate condition	Little control over size
	Microtissues of MSCs in chitosan-collagen matrix suspended in oil	[33]		
Microfluidics	PEGDA microgel containing single MSC or chondrocyte produced by emulsification in microfluidic device	[34]	Consistency Control the size of droplets	Low throughput Require rapid crosslinking materials
	Alginate microgel containing single MSC or pre-adipocyte cell	[35]		
	GelMA microparticles containing fibroblasts produced by emulsification in microfluidic device	[36]		
Cell electrospinning	Fibers of Matrigel containing mouse neuroblastoma cell produced by electrospinning	[37]	Guide cell aligned Efficient and fast nutrient exchange	Inhomogeneous cell density Low mechanical strength
	Fibers of alginate containing myoblast cells produced by cell electrospinning	[21]		
Micromolding	ECM with micro vasculature structure molded by PDMS chips	[38]	Fabricate complex structure High precision	Require precise template Laborious
	Vasculature network on 3D PDMS chips	[39]		
	PLGA 3D microparticles assembled in a layer-by-layer sinister process	[40]		
Bioprinting	Nanofibers of peptide amphiphiles containing fibroblasts and ADCSs	[41]	Automated Controllability High fidelity	Damage to cells Require printable biomaterials
	Vascular conduits of gelatin and alginate produced by microfluidic bioprinting	[42]		
	GelMA embedded with designed vascular network of HUVECs and fibroblasts	[24]		
	GelMA microgels with customized size and shape	[43]		
	Liver models of GelMA containing hepatocytes and human stellate cells with micro channels	[44]		

hMSC, human mesenchymal stem cell; ISC, intestinal stem cell; hiPSC, human induced pluripotent stem cell; ECM, extracellular matrix; ADSC, adipose-derived stem cell

### 3D culture

Organoids are 3D cultured miniature systems that are similar with living tissue in the aspect of function and structure [45]. Generally, organoids are made up of human adult stem cells or pluripotent stem cells growing in extracellular matrices. Many environmental cues, including stiffness of substrate, pattern of surface, growth factor, cell adhesive ligands, modulate the growth and differentiation of organoids [17, 28, 46]. Besides being used as a research tool in cell biology, organoids are also used as building blocks for tissue engineering. They can spontaneously grow and differentiate and subject to environmental cues at the same time [45]. However, limited productivity and poor mechanical property make organoids difficult to assemble, impeding their application in subsequent process of modular tissue engineering.

### Emulsification

When stirring an aqueous phase liquid with an organic phase, emulsification occurs and leads to aqueous liquid encapsulated by oil phase, which aggregates into droplets. This phenomenon is utilized to fabricate building blocks for tissue engineering. Gelatin, collagen, chitosan are the most commonly chosen ‘aqueous phase’ in producing cell-loaded building blocks [47]. The organic phase or oil phase is usually paraffin oil, which is safe and economic. After formation of miniature droplets, those droplets need to be crosslinked. For alginate, droplets are solidified in calcium or magnesium ion solution. For photocurable hydrogel, droplets are crosslinked under ultraviolet. The droplets, dubbed as micro-tissues in some studies, can be applied to construct glands [32] and solid organs [33]. In general, emulsification is an easy and moderate method to produce building blocks for tissues, but it can only fabricate spherical shaped microgels and the sizes are not controlled.

### Microfluidics

Microfluidics is a technique of manipulating the fluid flows in channels smaller than 1 mm. In microfluidics system, aqueous phase flows in the main channel and organic phase is injected to the channel at certain points, where emulsification takes place and particles form [48]. Piezoelectric injection and mechanical injection are alternative approaches of forming particles [49]. The size of particles is determined by several factors in microfluidics, including degree of injection angel, geometry of the intersection, viscosity of aqueous phase, velocity of the flow [34]. Photo-curable hydrogels such as PEG diacrylate (PEGDA) and gelatin methacryloyl (GelMA) are widely used in microfluidics because they can be quickly crosslinked by ultraviolet before piling up in the collecting container. Given the capacity of control the fluids precisely, microfluidics is suitable for encapsuling cells or other agents. Mao et al. used microfluidics to encapsulate single cell into alginate particles, which prolonged the effective period of transplanted cells in lung [35].

### Cell electrospinning

Electrospinning is a fabrication technique of projecting polymer solution under high-voltage electric field to fabricate ultrafine fiber. It is applied in the production of nanomaterials of various polymers [50]. Recently, electrospinning cell-laden biomaterials has been reported, and homogeneous cell distribution and high cell-viability are achieved [37]. In cell-laden electrospinning, cells are aligned along the fiber, mimicking structure of muscles or nerves [21]. Gelatin, alginate and collagen are mostly used in cell electrospinning because of biocompatibility. However, mechanical performance of cell electrospinning fibers is relatively poor and not comparable with targeted tissues. Besides, cell density of electrospinning fibers are usually inhomogeneous due to disturbance of cell-laden solution during fabricating process [51].

### Micromolding

Produced by lithography and microcontact technique, polydimethylsiloxane (PDMS) with intricate pattern provides an ideal template for molding biomaterials into building blocks for modular tissue engineering. The templates are accounted as ‘PDMS stamp’. Most building blocks manufactured by micromolding are regular-shaped like spheres, hemispheres, cylinders and rods. These simple models are generally used for single cell culture in hydrogels [52, 53]. ‘PDMS stamp’ with complex pattern has also been reported. Zhang et al. created ‘Angio-chip’, a biodegradable scaffold emulating pattern of vasculature on lithographic ‘PDMS stamp’ with micro channels. The channels of Angio-chip were later seeded with endothelial cells and they possessed properties of vessels—perfusability, permeability and sprouting [38]. Shin et al. invented a lithography technique based on the pyrolysis effect of consecutive laser to manufacture patterned PDMS with higher speed. They successfully used their technique to produce ‘vasculature-on-a-chip’ and ‘skin-on-a-chip’ [39]. But micromolding technique largely depends on manual work and the process of decoupling template and materials often cause rupture. When it comes to stacking a large amount of building blocks, this technique is laborious and error-prone.

### Bioprinting

Bioprinting allows precise control over the placement of materials and cells within constructs. Based on different mechanisms, there are three types of bioprinting: inkjet, extrusion-based and light projection. All of them can be applied in fabrication of building blocks for modular tissue. Inkjet printing sprays droplets of bioinks by vaporizing solution or mechanically squeezing the gel to force out drops. Extrusion printing harnesses the shear-thinning property of non-Newtonian liquid that bioinks flow through the nozzle under pressure and solidify into filaments after printing. Light projection printing uses ultraviolet to solidify photocurable hydrogelinto desired shape [54].

Inkjet printing deposits cell-laden droplets in arranged position. Droplets made in this technique are not stable. Additional polymerization process, such as photo-crosslinking, ion-exchange reaction, is required. Some studies aim to introduce new mechanism of polymerization in this process to enhance stability of droplets. Li et al. applied DNA hybridization in an inkjet bioprinting system so that the DNA-contained hydrogel can stabilize within second after they leave the snozzle [55]. Compared with the other two types of bioprinting, inkjet printing causes less damage to cells and cell viability is usually above 80%, but the printed modules are not mechanically strong.

Extrusion-based printing squeeze out filaments of bioinks and filaments are fundamental units in this technique. Wang et al. took advantage of the filament units to create dual-layered hollow conduits, which resemble the structure of veins and arteries. The printed conduits exhibited similar mechanical properties, perfusability, barrier performance with vessels [42]. Extrusion-based printing system is flexible to be modified and stable during the printing process, but the force exerted on cells is detrimental to their viability. To integrate advantages of inkjet bioprinting and extrusion-based bioprinting, Liu et al. used the latter to construct basic hydrogel strands firstly, then put droplet arrays on the scaffold through the former [56].

Light projection printing is an emerging technique. Attributed to precise light projecting device, it can create building blocks for tissues with high resolution. Yang et al. solidified PEGDA hydrogel within resolution of 100 micron. They successfully made irregularly shaped models with vertices, for facilitating the up-coming assembly [56]. In spite of ultraviolet applied in the manufacturing process, some studies manifest that light projection bioprinting can yield high cellular viability [16, 43]. Xie et al. designed a composite bioink of ECM particles and GelMA with satisfactory printability and cellular viability. The printed scaffold containing chondrocytes showed good performance on chondral regeneration in vitro and in vivo [57].

## Assembly of building blocks through bioprinting

After building blocks are manufactured, they need to be assembled into an integral bulk. Mechanism of assembly process can be categorized into: self-assembly, remote assembly and directed assembly [5]. Self-assembly is a thermodynamically driven process, referring chemical binding, physical interactions, biological adhesion or morphological recognition [58]. The condition where self-assembly occurs is moderate and generates little harm to cells. However, this spontaneous process lacks controllability. It is hard to stack building blocks into desired pattern and size under self-assembly mechanism. Remote assembly makes use of force field to manipulate building blocks into prearranged positions. In this method, building blocks are precisely assembled under acoustic fields, magnetic fields or optical fields [59, 60]. Yet this technique is confined in limited scenarios because of needing force fields and difficulty in scaling up adequate amount of building blocks.

Directed assembly requires energy input to connect building builds and thus it can achieve higher degree of customization than self-assembly and remote assembly. Directed assembly can be highly manual or automated, respectively in means of packing, bundling, stacking or bioprinting. Manual work aims at piling up building blocks to augment function of engineered tissues [61–63], which is limited in the quantity of assembled building blocks and the precision of manufacturing. Bioprinting, the automated form of directed assembly, is more advantageous in constructing spatially organized tissues. The three types of bioprinting mentioned above are applied in assembly process as well, namely inkjet bioprinting, extrusion-based bioprinting and light projection bioprinting (see Table 3). In this section, we depict the process of assembling building blocks using these three types of bioprinting. And we describe the factors effecting the printing process and strengths of each type of bioprinting.

**Table 3:** Bioprinting used to assemble building blocks

Bioprinting technique	Printing mechanism	Bioinks	Building blocks	Cell viability (optimal condition)	Printing resolution	References
Inkjet bioprinting	Valve-based printing approach	1.5% (w/v) alginate hydrogel containing hiPSC or hESCs-derived HLCs	Droplets for liver tissues	Around 55% for 23 days	100 μm	[64]
	Micro-droplet jetting including microfluidic chips	20% (w/v) gelatin hydrogel containing single human breast cancer cell or HUVEC	Droplets for cancer disease models	87% after 7 days	10 μm, 0.1nL	[56, 65]
	Oil-immersed nozzle printer into a lipid-in-oil bath	8:1 (v:v) mixture of 15 mg/ml ULGT-agarose to Fmoc-dipeptide solution containing HEKs or oMSCs	Droplets for cartilage-like tissues	91% after printing	< 200 μm, 1nL	[66]
	Ejection within a gentle acoustic field	0.5 wt% agarose hydrogel containing mESC, RAJI, HL-1, 3T3 or AML-12	Droplets encapsulating single cell	> 89.8% after printing	around 37 μm	[67]
Extrusion-based bioprinting	Extrude with gel-loaded syringes on the heated stage	13 wt% F127 and 6%wt alginate hybrid gel containing hMSCs	Filaments for cartilage or bone tissues	Around 83% after 7 days	400 μm	[68]
	Extrude layer-by-layer and dual-crosslink	5-20 wt% methacrylated Ad-HA and CD-HA	Filaments for guiding cell growth	–	100–500 μm (adjustable)	[69]
	Freeform reversible embedding of soft hydrogels	17.5 wt% PEG-αMA hydrogel containing HPAAFs	Filaments for PAH research models	Around 60% after 21 days (300 μm)	300 μm or 500 μm	[70]
	Extrude into perfusable silicone chips	10 mg/mL fibrinogen, 7.5 wt% gelatin, 1 wt% transglutaminase containing hMSCs and hNDFs	Filaments for vasculature	90% after printing	200 μm	[71]
	Temperature-controlled extrusion and post-printing crosslinking	5%/7.5%/10% (w/v) gelatin, 1% (w/v) alginate containing ESCs	Filaments for cell viability test	> 90% after printing	150 μm	[72]
	Extrude through a printhead with seven branches	5 wt% GelMA and 1 wt% alginate containing HDFs, HepG2, hMSCs or HUVECs	Filaments for gradient structures	Around 80% after 7 days	100–200 μm	[73]
	Extrude through coaxial nozzles with two inlets	1% (w/v) alginate and 1% (w/v) gelatin containing Min6 and HepG2	Filaments for perfusable network	Around 80% after 7 days	50 μm	[74]
	Compact bioink in syringe reservoir and extrude through nozzle	10% (w/v) collagen containing cardiac spheroids of hiPSC-CM and hDNFs	Elongated microtissues for cardiac tissues	Around 90% after 7 days	600 μm	[22]
Light projection bioprinting	Photo-crosslinking of methacrylate via image projection	20 wt% PEGDA containing HUVECs or fibrin gel containing hepatocytes	Vascularized alveolar or hepatic units	–	5pL	[4]
		2.5% (w/v) GelMA and 1% (w/v) GMHA containing hiPSC-induced HLCs	Hepatic models	65% after 7 days	< 200 μm	[16]
		10% (w/v) GelMA containing fibroblasts	Customized shape and size microgel	> 90% after 48 h	10 μm	[43]
		10% (w/v) GelMA and 10% (w/v) cartilage microtissues containing chondrocytes	Cartilage microtissues	> 90% after 20 days	200 μm	[57]
		2.5% (w/v) GelMA, 1% (w/v) HA containing HUVECs and fibroblasts	Vascularized tissues at microscale	> 80% after 7 days	20 μm	[75]
	Photo-crosslinking of norbornene via image projection	3–9 wt% PEG8NB along with PEG4SH	Micro scaffolds for cell culture	–	50 μm	[76]

hESC, human embryonic stem cell; HUVEC, human umbilical vein endothelial cell; ULGT-agarose, ultra-low-gelling-temperature agarose; HEK, human embryonic kidney cell; oMSC, ovine mesenchymal stem cells; mESC, mouse embryonic stem cell; RAJI, human Raji cell (a B-cell line); HL-1, HL-1 cardiomyocytes; AML-12, AML-12 hepatocytes; PEG-αMA, poly(ethylene glycol) alpha methacrylate; HPAAF, primary human pulmonary artery adventitia fibroblasts; PAH, pulmonary arterial hypertension; hDNF, neonatal dermal fibroblast; Min6, Mouse insulinoma 6; HepG2, Hepatocellular carcinoma; PEG8NB, Eight-arm PEG − norbornene; PEG4SH, Four-arm PEG-thiol; hiPSC-CM, human induced pluripotent stem cell

### Inkjet bioprinting assembles droplets

In inkjet bioprinting system, formation and assembly of building blocks for tissue engineering are integrated into a successive process, given that inkjet bioprinting yields droplets of bioinks which can be directly assembled. In contrast to extrusion-based or light projection printing, there is no external factors to confine the shape of bioinks in inkjet printing. Therefore, bioinks applied in this technique are mostly self-assembled. The droplets of bioink, namely the building blocks in inkjet bioprinting, are positioned according to the 3D model by the printing device and assembled into the whole bulk. The strength of inkjet printing is its good biocompatibility. Inkjet bioprinting is nontoxic to cells and it has been investigated in printing various cells, including induced pluripotent stem cells (iPSCs), human embryonic stem cells (hESCs) [64], neurons [77], and cancer cells [78]. Hedegaard et al. designed a self-assembling biomolecule bioink, which was constituted by peptide and protein. In this composite bioink, fibroblast and adipose cells co-existed with viability higher than 80% [41]. However, low resolution is an inherent drawback of inkjet printing [79]. The shape of building blocks is not controlled and the droplets usually won’t stabilize at arranged position. Reducing the volume of droplets to picolitre can improve printing resolution. Liu et al. and Mi et al. yielded jet printing droplets at the scale of picolitre through the vibration of viscous and inertial force. The resolution of assembly was at micrometer and it realized the arrangement of single-cell array [56, 65]. Supporting bath surrounding the printed structure can stabilize droplets, thus enabling printing at higher resolution. Graham et al. used the lipid bath, a viable medium for printing aqueous droplets, to support inkjet printing of cell-laden hydrogel. Their system reached the resolution of one nanoliter and the assembled droplets stably connected [66].

Although inkjet bioprinting has been used for decades, it is still competitive in producing 3D structure with high cell viability and assembling natural derived biomaterials. More types of inkjet printing have been invented, such as thermally driven, vibration driven, valve-based, acoustic, which enable this technique to adapt to different biomaterials within a range of viscosity [43]. And most of them can print at higher speed, compared with extrusion-based and light projection bioprinting.

### Extrusion-based bioprinting assembles filaments

Extrusion-based printing is the most popular addictive manufacturing technique in academic research and industry nowadays. In this technique, bioinks are extruded under pressure through the nozzle and the building blocks of this bioprinting are filaments. The printing device moves in the point-to-point manner to assemble the filaments. Many natural and synthetic hydrogels can be bioinks for extrusion-based printing as long as they possess the property of ‘shear-thinning’, which means they tend to be liquid under shear force and tend to be elastomer when no force applied. Shear-thinning property of bioinks effects the placement and assembly of filaments. Therefore, some studies aimed to develop new method to confer bioinks the shear-thinning property. Highley et al. reported a pair of chemical groups, adamantane (Ad) and β-cyclodextrin (β-CD), to modify hyaluronic acid. These two groups form intermolecular noncovalent and reversible bonds rapidly when they encounter, thus reinforce shear-thinning property of the hydrogel [80]. The resolution of extrusion-based printing depends on the size of filaments, but the crosslinking after extruding also influences the fidelity of assembly. Armstrong et al. combined thermal reversible hydrogel Pluronic F127 and alginate to make a balance between printability and fidelity of bioprinting. Extruded Pluronic F127 to a heated stage enhanced the printability and resolution. The alginate allowed post-printing stability in CaCl_2_ solution to sustain the fidelity[68].

Filaments, as building blocks, are easier to manipulate and more stable at their arranged position than droplets. Regarding the shapes, they are commensurate with some specific tissues, for example the arteries and veins [8, 42]. Nevertheless, these fundamental units make it difficult to print 2D shapes like circles and arches. And detachment between layers can occur and leads to the collapse of the whole structure. Double crosslinked hydrogels, which can be further crosslinked after extrusion, is a solution to this problem. Ouyang et al. modified hyaluronan with Ad group and methacrylic group, creating a chemically crosslinked and photo-crosslinked hydrogel for extrusion bioprinting. After the bioprinting is completed, constructs were consolidated under ultraviolet, as the methacrylic groups on backbone makes the polymers photocurable [69].

In extrusion-based bioprinting, supporting bath can be employed to stabilize the assembly process. The component of supporting bath can be crosslinking reagent solution, self-healing hydrogel, or oil phase [25, 70]. The printed construct is supported in the bath and thus the risk of collapse decreases. Moreover, roles of supporting bath and bioink can be reversed, where supporting bath is cell-contained biomaterial for tissue regeneration and bioink is sacrificial for creating cavity or canal. Kolesky and Skylar-Scott printed sacrificial ink into bath of cell aggregates or cell-laden gelatin, creating space for epithelial cells[31, 71].

Owing to the damage of shear force on cells in bioinks and the long duration of printing process, extrusion-based bioprinting usually causes low cell viability [72]. To assuage the damage, core–shell nozzle is an alternative because the crosslinking reagent solution flowing through the central outlet allow the pressure decrease and not influence the formation of constructs [42, 74].

### Light projection bioprinting assembles cured layers

Light projection bioprinting has emerged as a promising technique to assemble biomaterials into a complex and delicate construct. Sheets of cell-laden bioinks which are crosslinked under ultraviolet projection are stacked layer by layer in this type of bioprinting. The successive layers are conjunct after photo-crosslinking and the whole structure is coherent. Compared with extrusion-based bioprinting, construct made by this technique is resistant to collapse. And the printing resolution is satisfying. In light projection bioprinting system, digital micromirror device (DMD), a precise optical instrument is essential to ensure resolution of bioprinting. It is reported that the finest resolution of light projection bioprinting is less than 10 μm [81]. Besides, UV absorbers, which are usually dyes such as tartrazine, can be added at low concentration to bioink to improve the resolution of bioprinting [4]. Zhou et al. harnessed light projection bioprinting to construct a scaffold with tiny cavities and channels for skin regeneration, promoting cell migration, proliferation and tissue regeneration [82]. Light projection technique can also assemble repetitive units efficiently. The layer-by-layer printing manner prints the repetitive units in one layer simultaneously according to the model design. Several studies have reported using light projection printing to recreate complex network of vessels and biomimetic liver lobule [16, 44, 75], which are difficult to fabricate through extrusion-based printing.

The assembly mechanism of light projection bioprinting relies on the crosslinking of photocurable bioinks. Bioinks for light projection bioprinting consist of photo-sensitive monomers or oligomers, photo-initiators (PIs), cells and solvent. The most common functional group of photocurable bioinks invented so far is methacrylic group, for example hyaluronic acid methacryloyl (HAMA), GelMA, PEGDA. They react with free radicals emitted by PIs under lights of certain wavelength. Other photo-sensitive groups, PIs and their corresponding bioinks have also been reported. Poly(ethylene glycol)-norbornene is a low-viscosity bioink for light-initiated crosslinking. Kim et al. have proved its printability and compatibility with cells [76]. Water-soluble PIs for Two-photon polymerization (2PP) were proven efficient in cell-laden gelatin, which facilitates utilization of 2PP in bioprinting and provide a possible way of bioprinting in situ without movement of bioinks [83, 84]. Other mechanisms of photo-polymerization have also been studied. Cationic reversible addition fragmentation chain transfer (RAFT) [85], dual-color photo-polymerization [86] have been proven that they can be applied in light projection printing. Yet whether they are suitable for bioprinting should be further investigated. Volumetric printing is an emergent bioprinting technique relying on precise stereomicroscopy. It can print in situ inside bulk of materials like 2PP but it does not require special PIs and it is compatible with most available photo-curable bioinks [87, 88].

Cell damage caused by high-intensity lights and cytotoxic PIs should be considered and assessed before printing cell-laden biomaterials. The concentration of PIs and exposure time of UV light should range between not being poisonous and not affecting printability. Therefore, a cell cytotoxicity assay is recommended to determine the optional concentration and exposure time for bioprinting. Huh’s research and Rouillard’s research provide valuable reference in this regard [89].

## Bioprinting tissues containing multiple components

Living organs are composed of various tissues, working together to maintain the function of organs. Engineered tissue is supposed to replicate this complexity. Multi-material bioprinting methods have been studied to create spatially organized tissues. They are useful in constructing vascularized structures, customized organs and stable constructs with high cell viability [90].

**Fig. 3:**
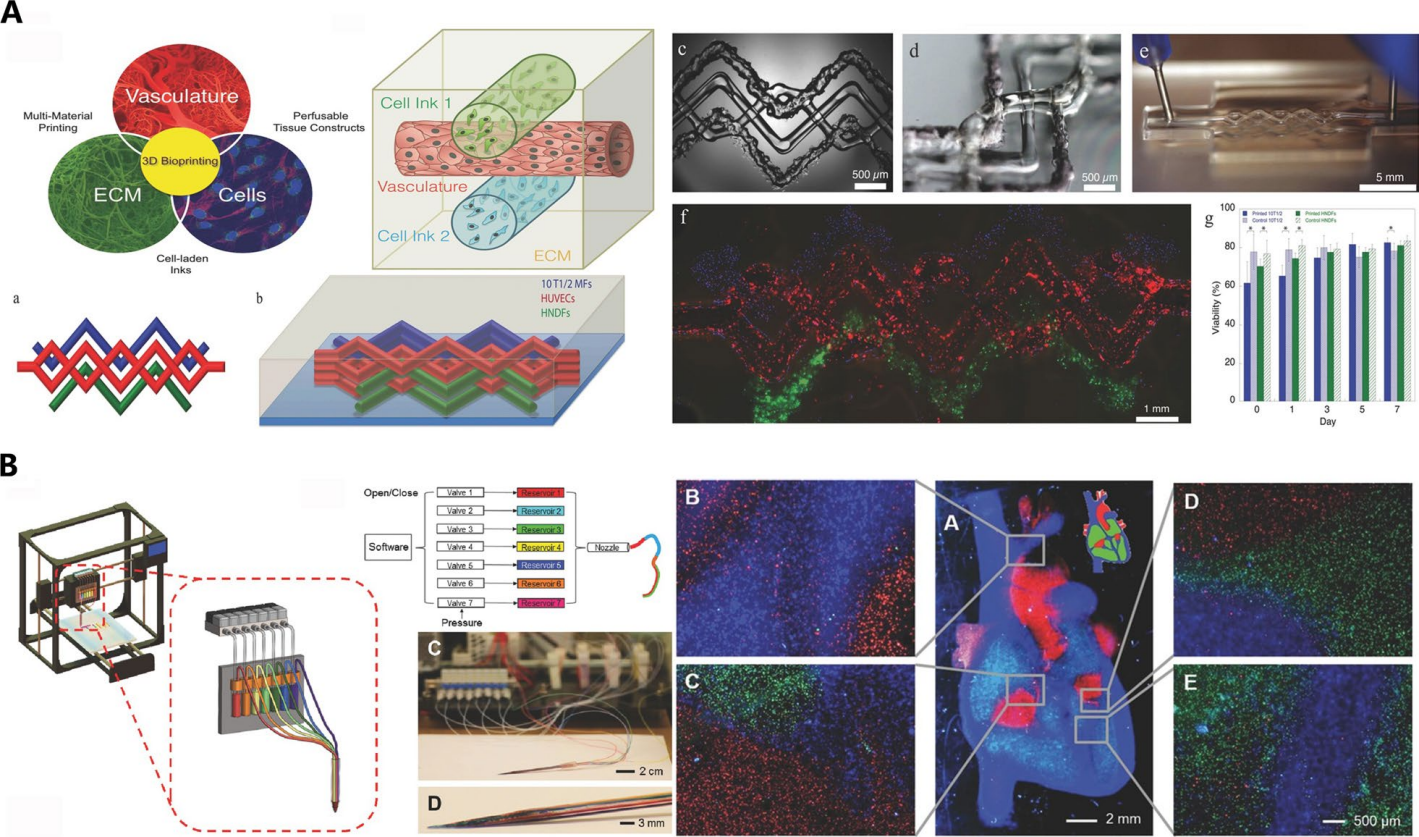
Multiple nozzles or reservoirs for multi-material bioprinting. **A** each nozzle corresponding to one component of tissues (Reproduced with permission. Copyright 2014, WILEY‐VCH Verlag GmbH & Co.); **B** different bioinks in each reservoir can be deposited in specific area (Reproduced with permission. Copyright 2016, WILEY‐VCH Verlag GmbH & Co.)

**Fig. 4:**
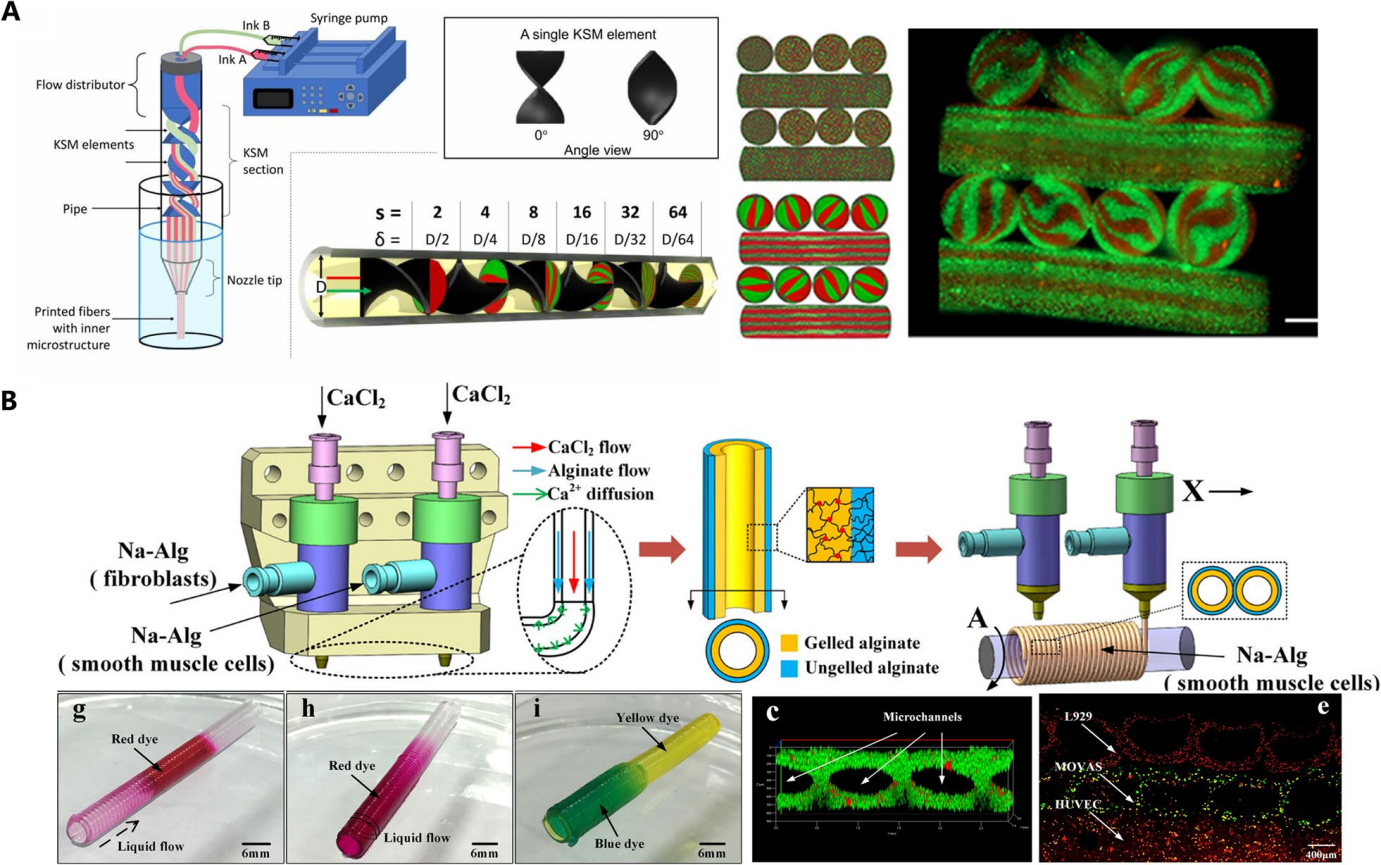
Novel structure in nozzle for multi-material bioprinting. **A** Using spiral valve to mix different materials (from Chávez-Madero C, de León-Derby MD, Samandari M, et al. Biofabrication. 2020;12(3):035023 Reproduced under the terms of the Creative Commons Attribution 4.0 license.); **B** using co-axial nozzle to print different bioinks simultaneously (Reproduced with permission Copyright 2017, American Chemical Society)

Nozzle switch or deployment of multiple nozzles is a solution to print bioinks of different composition in extrusion-based bioprinting. Kolesky et al. designed a 3D bioprinter with four printheads to produce heterogenous structure composed of different materials. The multi-printhead printer could embed fugitive inks between cell-laden materials to form a vasculature (Fig. 3A) [91]. Besides, other methods for multi-material bioprinting are also reported: single nozzle connected to multiple reservoirs (Fig. 3B) [73]; spiral valve which can cause advection (Fig. 4A) [92]; co-axial nozzle with different bioinks flowing (Fig. 4B) [93].

**Fig. 5:**
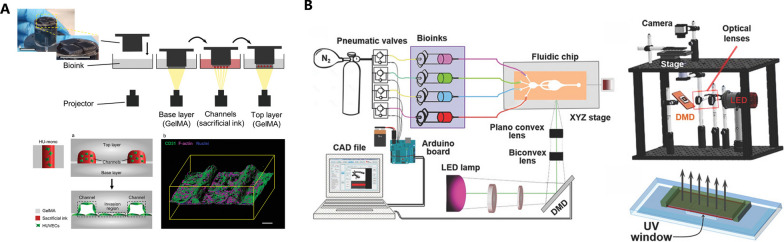
Transition of bioinks for multi-material DLP bioprinting. (**A**) changing the bioink container after printing specific layers (from Orellano I, Thomas A, Herrera A, et al. Adv Funct Mater. 2022;32(52):2208325. Reproduced under the terms of the Creative Commons Attribution- NonCommercial License.); **B** rapid switch of bioinks by a pneumatic-driven pump (Reproduced with permission Copyright 2018, WILEY‐VCH Verlag GmbH & Co.)

As for light projection bioprinting, transition of container of different bioinks is required. For example, Orellano et al. transformed containers during the printing procedure to fabricate a triple layer tissue through light projection printing, where each layer consists of different cells and bioinks (Fig. 5A) [94]. Similarly, rapid exchange of bioinks in container is also an option for multi-material projection bioprinting (Fig. 5B) [95]. But if more layers need to be printed to construct tissues, flexible and automated instrument like robotic arms can be applied [96].

The design of multiple components constructs can be conducted by researchers in computer aided design software such as Unigraphics NX, Solidworks, AutoCAD, according to anticipated function of tissues or structure of living tissues. However, it is also significant to construct clinically relevant tissues [97]. Clinical data obtained from patients’ CT or MRI examination can be the source and reference for the design of engineered tissues [98].

## Conclusion

The bottom-up strategy in tissue engineering provides an approach to mimic living tissues. The fabrication and assembly of building blocks confer artificial tissues with improved cell distribution, cell viability, vascularization and tissue integration. Several studies involving building blocks of tissues have illustrated the effectiveness of ‘bottom-up’ [16, 19, 22, 23, 38]. In this review we focus on the techniques for fabrication and assembly of building blocks. Although in these two steps of ‘bottom-up’ strategy, many methods can be harnessed but they vary in capacity to control the shape of building blocks and position the building blocks. Bioprinting is a recommended techniques for both the fabrication and assembly of building blocks. Many bioinks have also been developed for different types of bioprinting including inkjet, extrusion-based and light processing bioprinting. As for the limitation, bioprinting and other fabrication methods for building blocks can only process the biomaterials of the artificial tissues. Even if the processing and structure of materials can influence the cells, the effect is mostly indirect and has some extent of stochasticity.

## Discussion and prospects

Living tissues are physiologically complex and to mimic the physiological complexity is one of the challenges for tissue engineering. The goal of ‘bottom-up’ design strategy is aligned with solving this problem. The fabrication of building blocks can recreate the microstructure of tissues and organs. Assembly of building blocks can realize the spatial organization of living organs. ‘Building blocks’ are the center of ‘bottom-up’. The automation and customization of fabrication can be achieved by bioprinting. Therefore, bioprinting is a recommended option for the bottom-up strategy. Both fabrication and assembly of building blocks can be executed by bioprinting with precision and robustness. Bioprinting has been extensively studied in this field and novel bioprinting methods, for example volumetric bioprinting [86, 99], have been proposed.

Development of bioinks is critical to the improvement of bioprinting. In addition to extant bioinks, bioinks which are both mechanically strong and biocompatible are needed. Besides, cells in bioinks can be modified for specific function. For example, membrane functionalization is applied to augment adhesion and infiltration of cells [100–102]. Furthermore, gene editing, through lentivirus, plasmid transfection, CRISPR/Cas9 or other approach, can be harnessed to produce cells with desired function in the construction of artificial tissues.

Creating large-scale tissues or organs which are clinically relevant is still challenging so far. The maximum quantity of building blocks that can be assembled is limited. Building blocks are supposed to be more stable and the speed of bioprinting is supposed to increase. In addition, engineered tissues should be able to survive in vitro and in vivo. Therefore, producing constructs of multiple types of tissue is important and there is supposed to be strategy for vascularization of artificial tissues. Mechanical innovations of bioprinting in the future may solve these problem mentioned above [88, 103]. Now 4D structures, which can reshape or reorganize along with time, have been reported. 4D structure can be attained through bioprinting by printing layers of concentration gradient [104, 105] or introducing condition sensitive particles [106]. Owing to time-dependent changing property, 4D structures might simplify the fabrication and facilitate assembly process. In the future, there will be more studies focusing on 4D bioprinting.

Bottom-up strategy has shown its efficacy in constructing functional artificial tissues. We think that ‘bottom-up’ will still be one of the cardinal principles in tissue engineering in the future. Although many challenges remain, we anticipate that new technology will arise and remarkable progress will be made.

## Data Availability

This review article involves no experimental data. References are listed in the end of this article.
